# Evaluating the efficacy of sequential biologic therapies for rheumatoid arthritis patients with an inadequate response to tumor necrosis factor-α inhibitors

**DOI:** 10.1186/ar3249

**Published:** 2011-02-16

**Authors:** Regina Rendas-Baum, Gene V Wallenstein, Tamas Koncz, Mark Kosinski, Min Yang, John Bradley, Samuel H Zwillich

**Affiliations:** 1 QualityMetric Inc., Outcomes Insight Consulting Division, 24 Albion Road, Lincoln, RI 02865, USA; 2 Pfizer Clinical Development and Medical Affairs, 50 Pequot Avenue, 6025-B3206, New London, CT 06320, USA; 3 Pfizer Clinical Development and Medical Affairs, 235 East 42nd Street, New York, NY 10017, USA

## Abstract

**Introduction:**

The long-term treatment of rheumatoid arthritis (RA) most often involves a sequence of different therapies. The response to therapy, disease progression and detailed knowledge of the role of different therapies along treatment pathways are key aspects to help physicians identify the best treatment strategy. Thus, understanding the effectiveness of different therapeutic sequences is of particular importance in the evaluation of long-term RA treatment strategies. The objective of this study was to systematically review and quantitatively evaluate the relationship between the clinical response to biologic treatments and the number of previous treatments with tumor necrosis factor α (TNF-α) inhibitors.

**Methods:**

A systematic search was undertaken to identify published, peer-reviewed articles that reported clinical outcomes of biologic treatment among RA patients with an inadequate response to TNF-α inhibitors. Data were systematically abstracted. Efficacy rates were estimated for groups of patients who differed in the number of prior TNF-α inhibitors used. End points included American College of Rheumatology (ACR)-, European League Against Rheumatism (EULAR)- and Disease Activity Score 28 (DAS28)-based response criteria.

**Results:**

The literature search identified 41 publications, of which 28 reported biologic treatment outcomes for RA patients with prior exposure to TNF-α inhibitors. Seven publications reported outcomes obtained in randomized clinical trials, while the remaining consisted of observational studies. The likelihood of responding to a subsequent biologic treatment decreased as the number of previous treatments with TNF-α inhibitors increased for six of the seven response criteria examined.

**Conclusions:**

For patients with prior exposure to TNF-α inhibitors, the likelihood of response to subsequent treatment with biologic agents declines with the increasing number of previous treatments with TNF-α inhibitors.

## Introduction

The chronic nature of rheumatoid arthritis (RA) and its progression over time in spite of a variety of treatment options implies that long-term treatment will most often involve a sequence of therapies. The optimal therapeutic sequence strategy will be determined largely by the patient's response to therapy and by disease progression, as well as detailed knowledge of the role of different therapies along treatment pathways. Thus, understanding the effectiveness of different therapeutic sequences is of particular importance in the evaluation of long-term RA treatment strategies.

There are three main drug classes commonly used in the treatment of RA: nonsteroidal anti-inflammatory drugs (NSAIDs), corticosteroids and disease-modifying antirheumatic drugs (DMARDs). Several studies [1-3] have provided evidence that early treatment with DMARDs results in superior clinical and radiological outcomes. Two main classes of DMARDs are available for the treatment of RA: synthetic DMARDs and biologic DMARDs. Oral administration, lower cost and greater prescriber familiarity support the use of synthetic DMARDs as a first-line strategy. Biologic DMARDs, most often in combination with synthetic DMARDs, are generally reserved for the treatment of patients with moderate to severe RA who have had an inadequate response or have developed toxicities to synthetic DMARDs [4].

A review of 16 clinical practice guidelines and 20 consensus statements on RA treatment revealed that while tumor necrosis factor (TNF)-α inhibitors were consistently recommended for patients with active RA and a history of inadequate response to synthetic DMARDs [5], the management of patients who stopped an initial TNF-α treatment because of lack of initial response, loss of initial response or side effects continues to be the subject of much debate, and guidelines for patient management are nearly absent. Despite the lack of guidelines, it is estimated that upon encountering an inadequate response or side effects with a TNF-α inhibitor, over 90% of rheumatologists in the United States switch patients to a different TNF-α inhibitor [6].

Estimates of efficacy rates of TNF-α inhibitors may depend on a number of factors, including patient characteristics, such as disease duration, prognostic factors, number of previously failed DMARDs and disease activity, as well as the dose of TNF-α inhibitor and the designs of the studies from which they were obtained. Despite some variation attributable to these factors, estimates derived from randomized, controlled trials (RCTs) suggest that between 40% and 50% [7] of RA patients treated for at least 6 months with one of the three first-generation TNF-α inhibitors (etanercept, adalimumab and infliximab) failed to achieve the American College of Rheumatology 50% (ACR50) improvement criteria [8], while the results from a large, registry-based study [9] indicated that over 70% of these patients fail to achieve Disease Activity Score 28 joint count (DAS28)-defined "remission" (DAS28 <2.6).

Although the efficacy of TNF-α inhibitors in patients who are naïve to biologic treatment has been evaluated in multiple studies [10-12], evaluating the efficacy of these drugs in patients who have already experienced an inadequate response to a TNF-α inhibitor poses greater methodological challenges. One key aspect of evaluating the efficacy of sequential TNF-α therapy is to determine whether the probability of responding to a TNF-α inhibitor depends on the results of prior treatment with these drugs. Early evidence from small observational studies suggested that a significant proportion of patients who had an inadequate response to an initial TNF-α inhibitor benefited from subsequent treatment with an alternative TNF-α inhibitor [13-15]. Recent data derived from registries, however, have suggested that the response rates of patients switching to a second or third TNF-α inhibitor are often lower than the response rates of patients to the first TNF-α inhibitor [16,17]. Moreover, the broader question whether it is more effective to switch to another mechanism of action or to use a second TNF-α inhibitor after the patient has had an inadequate response to a first one has not been formally addressed.

In the present study, several biologic treatment options currently available to RA patients with an inadequate response to an initial TNF-α inhibitor were evaluated using evidence gathered from published reports. We undertook a systematic review of published, peer-reviewed studies that reported clinical outcomes of biologic treatment among this group of patients. Our study expands on previously published reviews in two ways: first, information on efficacy rates of newer biologics with different mechanisms of action among patients with an inadequate response to TNF-α inhibitors was also included and results were examined separately for TNF-α inhibitors and other biologic DMARDs; second, a quantitatively based evaluation of the relationship between response to biologic treatment and the number of failed TNF-α inhibitors was undertaken by summarizing the results of published studies. Within the limitations of the existing data, potential effect-modifying factors, such as study design and treatment duration, were also examined. A secondary objective of this study was to determine whether clinical response to a subsequent TNF-α differed by reason for discontinuation.

## Materials and methods

### Search strategy

A search was carried out in the PubMed database using each of the following search terms as keywords or text words: "golimumab," "adalimumab," "infliximab," "etanercept," "abatacept," "rituximab," "anakinra," "tocilizumab," "certolizumab pegol," "anti-TNF," "TNF-antagonist," "TNF-inhibitor," "biologic*" in combination with "switch*" or "sequential therapy" or "therapy interchange," and "rheumatoid arthritis." Brand names of biologics were also used for each of the drugs cited above. The search was restricted to the English language and had an end date of 31 December 2009. The reference lists of selected review publications were further examined to identify any studies that were not captured by our search.

Articles were included in the analyses if the publications reported any quantitative clinical and/or health-related quality of life outcomes for RA patients previously failing one or more TNF-α inhibitors. Studies with fewer than 20 participants were excluded.

### Database development

The characteristics of each study were recorded, including the study design and major findings. Disease duration, age, sex distribution, duration of treatment, duration of washout period (if reported), concomitant use of methotrexate (percentage of patients within the group), dose of biologic drug and all clinical and quality-of-life measures were recorded for each group of patients on the basis of the total number of biologics that had been tried at the time the outcome was measured. Studies differed with respect to the way in which washout periods were reported. For this study, washout periods were noted in the following manner: (1) if the mean or median was reported (the median was preferred if both were reported) for the time elapsed between the last dose of prior treatment until the first dose of subsequent treatment, this value was recorded; and (2) if no summary statistic for the washout period was reported, the minimum washout period required per study protocol was recorded. For RCTs in which different doses of biologic DMARDs were administered, efficacy estimates based on all study arms were included. The sensitivity of the results to this parameter was assessed in the analyses.

Some studies reported outcomes of multiple switches for the same group of patients, so the same group of patients might have contributed to more than one combination of outcome measures and number of biologics tried. A few studies did not report results disaggregated by the actual number of prior TNF-α inhibitors tried and reported only the results of the biologic under study for subjects with an inadequate response to at least one TNF-α inhibitor (in these cases, the number of biologics under study was recorded as 2+).

Several studies did not allow for within-study evaluation of differences in clinical or health-related quality-of-life outcomes across groups differing in the number of previous TNF-α inhibitors used. For these studies, the results of various outcome measures were reported for a single comparison group.

### Efficacy estimates

All estimates were evaluated for each combination of measure and biologic number (that is, first, second, and so on), as well as for relevant subgroups. All estimates were evaluated as weighted averages using sample size as the weight in the following formula:

$$Rate_{j}=\sum_{i=1}^{n_{i}}\frac{\left(SS_{i}\cdot {\text{Rate}}_{\text{i}}\right)}{\text{N}},$$

where Rate_*j *_represents the average response rate for measure *j*, *i *indexes the group, *n*_*i *_is the sample size for the *i*th group and *N *is the combined sample size of all groups.

The main focus was on estimating the efficacy rate on the basis of each of the main response criteria reported in the studies identified by our review across the number of previously failed biologics. Nevertheless, some of the publications included in the current study also reported efficacy rates associated with a first trial of TNF-α treatment. Weighted estimates were also evaluated for this group of patients and served as a further check of how the values obtained in the current study compared to published rates. Estimates were also evaluated within the following subgroups: type of study (observational study versus RCT), duration of follow-up (<6 months versus 6 months or longer), type of biologic (TNF-α inhibitor versus other) and reason for discontinuation (lack of response, loss of response or intolerance).

## Results

### Characteristics of biologic treatments for RA

Table 1 presents a brief overview of current biologic DMARDs, including their brand names, dates of approval by the Food and Drug Administration for the treatment of RA, mode of action and schedule of administration.

**Table 1:** Biologic DMARDs for the treatment of RA^a^

Generic drug name (brand name, year of FDA approval)	Structure and mechanism of action	Mode and frequency of administration
TNF-α inhibitors		
Infliximab (Remicade, 1999)	Chimeric monoclonal antibody that binds to TNF-α and blocks its interaction with cell surface receptors	Intravenous infusion every 8 weeks
Etanercept (Enbrel, 1998)	Soluble human fusion recombinant protein that binds to TNF-α and blocks its interaction with cell surface receptors	Subcutaneous injection weekly or twice weekly
Adalimumab (Humira, 2002)	Recombinant human monoclonal antibody that binds to TNF-α and blocks its interaction with cell surface receptors	Subcutaneous injection every 2 weeks (or weekly if methotrexate is not taken concurrently)
Golimumab (Simponi, 2009)	Human monoclonal antibody that binds to TNF-α and blocks its interaction with cell surface receptors	Subcutaneous injection monthly
Certolizumab pegol (Cimzia, 2009)	Recombinant, humanized, pegylated Fab' of a monoclonal antibody that binds to TNF-α and blocks its interaction with cell surface receptors	Subcutaneous injection every 2 or 4 weeks, if dosed at 200 mg or 400 mg, respectively.
		
Other biologic DMARDs		
Abatacept (Orencia, 2005)	Soluble fusion protein that inhibits the costimulation of T-cells	Intravenous infusion every 4 weeks
Anakinra (Kineret, 2001)	Recombinant IL-1 receptor antagonist that inhibits the binding of IL-1 to its receptor, thereby allowing regulation of IL-1 activity	Subcutaneous injection daily
Rituximab (Rituxan, 2006)	Chimeric monoclonal antibody that binds to the cell surface protein CD20 and selectively depletes B-cells.	Intravenous infusion: two infusions separated by 2 weeks every 24 weeks or based on clinical evaluation
Tocilizumab (Actemra, 2010)	Humanized IL-6 receptor that inhibits the binding of IL-6 to its receptor, preventing IL-6 signal transduction	Intravenous infusion every 4 weeks

^a^DMARDs, disease-modifying antirheumatic drugs; Fab', fragment antigen-binding region; RA, rheumatoid arthritis; FDA, Food and Drug Administration; TNF, tumor necrosis factor; IL, interleukin.

### Study characteristics

On the basis of an abstract review, 41 publications were identified as potentially reporting clinical outcomes of patients who had switched to a second or subsequent biologic DMARD. Upon full review of the 41 publications, 28 were included in the study. The remaining 13 publications [16,18-29] were excluded for one or more of the following reasons: (1) the information reported was not relevant to the objective of our analysis, (2) quantitative results could not be extracted from the publication, and/or (3) the study sample did not include patients with an inadequate response to one or more TNF-α inhibitors. Our search did not uncover any information that pertained to the effect of certolizumab pegol or anakinra for the treatment of patients with an inadequate response to TNF-α inhibitors. Two [30,31] of the 28 publications included reports that contained analyses of patients with conditions other than RA. The percentage of RA patients was 80% and 95% across these two studies. Both studies were small, with a combined sample size of 93 patients. Of the 26 remaining publications, five were randomized trials (one was not placebo-controlled). One publication [32] reported results associated with different doses of tocilizumab (4 and 8 mg).

Key characteristics of the 28 [14,15,17,29-53] selected publications, including a brief description of their key findings, are presented as supplementary material (Additional file 1). The studies used in specific subgroup analyses are also identified.

### Outcome measures

The types of outcome measures reported across the 28 publications differed considerably. The most commonly reported efficacy measures were ACR-, European League Against Rheumatism (EULAR)- and DAS28-based response criteria. Health Assessment Questionnaire (HAQ) scores were also commonly reported, but in different ways across studies. In some cases, the publication reported mean values at baseline and posttreatment, while in other cases only the absolute or percentage change from baseline were reported. ACR-based response criteria were reported for all seven drugs, while most of the other measures were available for three or four drugs. Information on response rates across groups based on the number of previous TNF-α inhibitor treatments differed substantially by drug. The efficacy of etanercept, for example, although available across a number of response criteria, was explicitly reported only for patients with one previous TNF-α inhibitor treatment trial. In addition, some studies did not report the actual drug used and presented results aggregated over the three first-generation TNF-α inhibitors (adalimumab, etanercept and infliximab). On the basis of the greatest availability of data and relevance as markers of clinical response, the following efficacy measures were selected: ACR20, ACR50 and ACR70 rates; DAS28 low disease activity rates (DAS28 ≤3.2) [54]; DAS28 remission rates (DAS28 <2.6) [55]; and EULAR-based rates of moderate and good responses [56].

### Efficacy estimates based on number of previous TNF-α inhibitors

**Figure 1:**
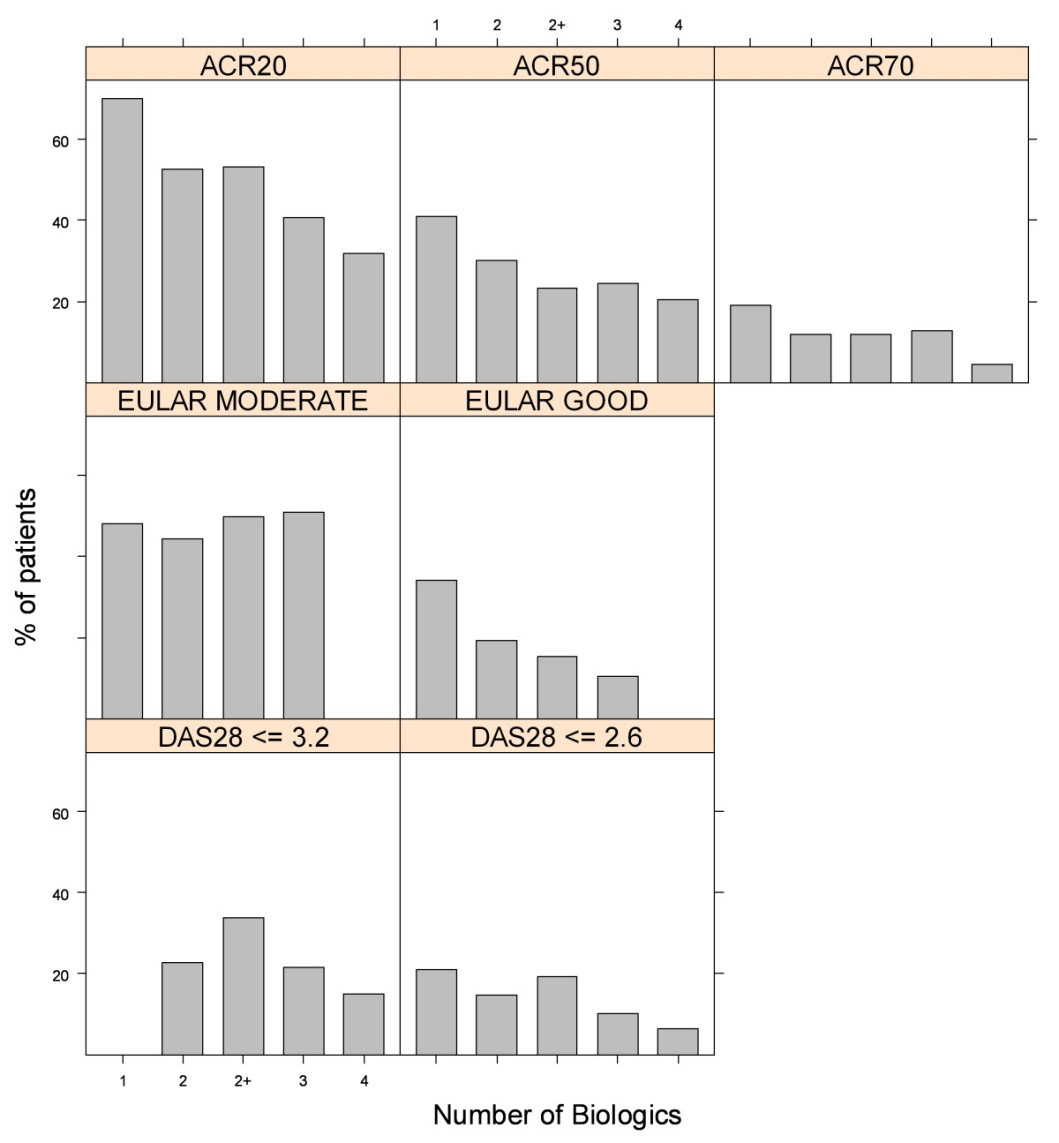
**Percentage of patients achieving a response according to biologic treatment number**. Bar graphs showing the percentage of patients achieving a response to any biologic disease-modifying antirheumatic drug (DMARD) according to criteria commonly used in rheumatoid arthritis (RA) patients, separated by number of biologic DMARDs to which the patients were exposed. With regard to the ACR categories, ACR20 means a 20% improvement in tender or swollen joint counts as well as 20% improvement in at least three of the following five criteria: patient assessment, physician assessment, erythrocyte sedimentation rate, pain scale and functional questionnaire. The ACR50 and ACR70 categories adhere to the same criteria, but for 50% and 70% improvement, respectively. ACR, American College of Rheumatology; EULAR, European League Against Rheumatism; DAS28, Disease Activity Score 28 joint count.

Average response rates by number of TNF-α inhibitors are shown in Figure 1 for ACR-, EULAR- and DAS28-based response criteria. The bar graphs in Figure 1 show that for six of the seven indicators examined, the likelihood of patient response to a subsequent biologic treatment decreased slightly in patients with a greater number of previous treatments with TNF-α inhibitors.

The main exception to the trend of decreasing likelihood of response was the association between EULAR moderate response rates and the number of previous TNF-α inhibitors. Upon close examination, the study characteristics do not appear to explain this difference (see Table 2). From among the 10 studies used to derive EULAR response rates, only three [33,36,45] reported these rates on the basis of the number of previous TNF-α inhibitors. For these three studies, within-study differences in good EULAR response rates consistently declined with increasing number of previous TNF-α inhibitors. However, for the same three studies, within-study differences in moderate EULAR response rates by number of previous TNF-α inhibitors did not show a clear trend. For example, on the basis of the results of the ReAct open-label trial [36], good EULAR response rates were 35%, 25% and 11% for the first, second and third TNF-α inhibitors administered, respectively, while for moderate EULAR responses, these rates were 49%, 53% and 51%, respectively. Thus, even within the same study, the relationship between moderate EULAR response rates and the number of previous TNF-α inhibitors administered was different from the relationship between good EULAR response rates and the number of previous TNF-α inhibitors used.

**Table 2:** Information used to evaluate the proportion of patients achieving a response according to common criteria used in RA studies^a^

Measure	Number of biologics used	Number of patients	Estimated response rate, %	Specific biologics used to derive response rate	Treatment duration range, months	Mean age range, yr	Mean RA duration range, yr	Reference sources
ACR20	1	5,762	70.0	ADA	6 to 12	53 to 56	11 to 16	[30,36,51]
	2	1,911	52.7	ADA, ETN, GLM, IFX, TCZ, TNF_2_	3 to 12	45 to 57	9 to 17	[14,15,17,30,32,34,36,37,51-53]
	2+	772	53.3	ABA, RTX	6 to 12	52 to 53	12	[39,41,42]
	3	339	40.6	ADA, GLM, TCZ, TNF_3_	3 to 6	51 to 58	11 to 15	[17,32,36,52]
	4	66	31.9	GLM, TCZ	4 to 6	51 to 54	11 to 13	[32,52]
ACR50	1	5,736	41.1	ADA	3 to 12	54 to 56	11 to 16	[36,51]
	2	1,699	30.1	ETN, TCZ, TNF_2_, ADA, IFX	3 to 12	45 to 57	9 to 17	[14,15,17,32,34,36,37,47,51]
	2+	1,078	23.5	GLM, ABA, RTX	4 to 12	52 to 55	9 to 12	[39,41,42,52]
	3	268	24.4	TCZ, TNF_3_, ADA	3 to 6	51 to 58	11 to 15	[17,32,36]
	4	44	20.5	TCZ	6	51 to 54	11 to 13	[32]
ACR70	1	5,736	19.1	ADA	3 to 12	54 to 56	11 to 16	[36,51]
	2	1,686	12.0	ADA, ETN, TCZ, TNF_2_	3 to 12	49 to 57	8 to 17	[5,17,32,34,36,37,47,51,53]
	2+	1,078	11.9	ABA, GLM, RTX	4 to 12	52 to 55	9 to 12	[39,41,42,52]
	3	268	12.7	ADA, TCZ, TNF_3_	3 to 6	51 to 58	11 to 15	[17,32,36]
	4	44	4.5	TCZ	6	51 to 54	11 to 13	[32]
DAS28 <2.6	1	5,711	21.0	ADA	3	54	11	[36]
	2	1,604	14.9	ABA, ADA, TNF_2_	3 to 6	53 to 56	12 to 14	[17,29,36]
	2+	331	19.2	TCZ	6	51 to 54	11 to 13	[36]
	3	496	10.2	ABA, ADA, TNF_3_	3 to 6	52 to 58	12 to 15	[17,29,36]
	4	200	6.5	ABA	6	56	-	[29]
DAS28 <3.2	2	1,219	22.7	ABA,TNF_2 _TNTNF_2_	3 to 6	55 to 56	8 to 14	[17,29,40]
	2+	331	33.7	TCZ	6	51 to 54	11 to 13	[32]
	3	376	21.6	ABA TNF_3_	3 to 6	56 to 58	15	[17,29]
	4	200	15.0	ABA	6	56	-	[29]
EULAR moderate	1	6,494	48.0	ADA, TNF	3 to 8	54 to 57	8 to 11	[33,36,45]
	2	1,854	44.4	ADA, ETN, TNF_2_	3 to 12	53 to 61	8 to 13	[35-38,43,45]
	2+	324	49.7	TNF, RTX	6 to 9	52	12	[33,39]
	3	120	51.0	ADA	3	52	12	[36]
EULAR good	1	6,494	34.0	ADA, TNF	3 to 8	54 to 57	8 to 11	[33,36,45]
	2	2,232	19.4	ADA, ETN, TNF_2_	3 to 12	53 to 61	8 to 14	[17,36-38,43-45,53]
	2+	324	15.3	TNF_2+_, RTX	6 to 9	52	12	[33,45]
	3	156	10.5	ADA, TNF_3_	3	52 to 58	12 to 15	[17,36]

^a^RA, rheumatoid arthritis; ACR, American College of Rheumatology; DAS28, Disease Activity Score 28 joint count; EULAR, European League Against Rheumatism. ABA, abatacept, ADA, adamlimumab, ANA, anakinra, ETN, etanercept, GLM, golimumab, IFX, infliximab, RTX, rituximab, TNF_*i*_, *i*th TNF-α inhibitor, TCZ, tocilizumab. ACR20 means a 20% improvement in tender or swollen joint counts as well as 20% improvement in at least three of the following five criteria: patient assessment, physician assessment, erythrocyte sedimentation rate, pain scale and functional questionnaire. The ACR50 and ACR70 categories adhere to the same criteria, but for 50% and 70% improvement, respectively.

Although less pronounced, a second departure from the general trend was the fact that the proportion of patients achieving low disease activity (DAS28 <3.2) and remission (DAS28 <2.6) was higher among patients in whom one or more TNF-α inhibitors had previously failed (bars labeled "2+" in Figure 1) than for patients with a single failed TNF-α treatment trial (bars labeled "2" in Figure 1). The source of the 2+ group value was a single RCT of tocilizumab [32] with a follow-up length of 6 months, while the estimated response rates regarding the second, third and fourth biologic treatments were obtained from observational studies, several of which had follow-up lengths of 3 months. In addition, in one of these observational studies, DAS28 was evaluated using C-reactive protein (CRP) level rather than erythrocyte sedimentation rate (ESR) level. Although CRP-based DAS28 scores are seen as a valid alternative to the more commonly used ESR-based DAS28 scores, there are reports that the former results in DAS28 values that are significantly lower [57,58], a finding that agrees with the graphs shown in Figure 1.

Studies differed on a number of factors, such as type of biologic, disease and treatment duration, which could have a strong influence on these estimates (see Additional file 1, Table S1). To ascertain how the overall estimates might have been affected by these factors, stratified estimates were also evaluated.

### Efficacy estimates stratified by type of biologic drug (TNF-α inhibitors versus other biologics)

**Figure 2:**
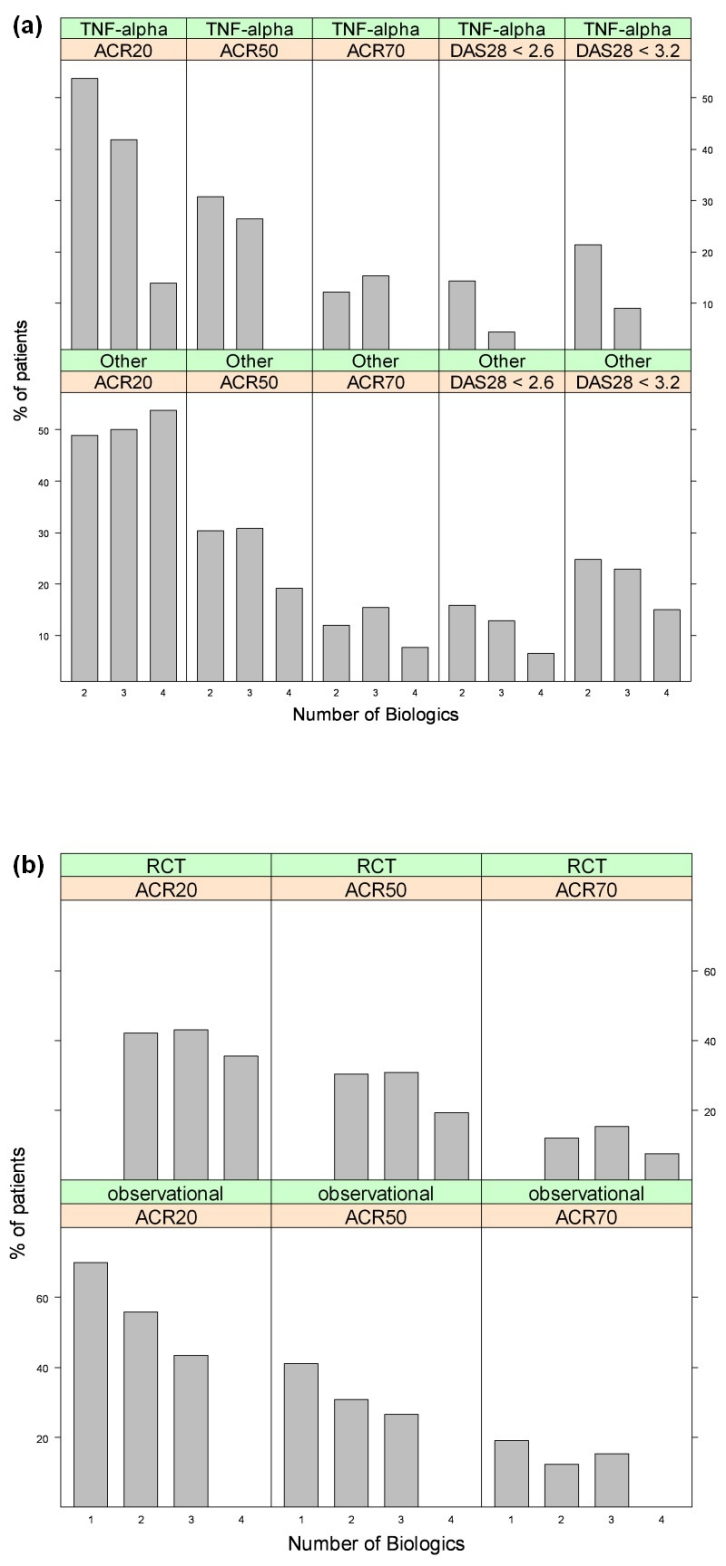
**Percentage of patients achieving a response by biologic type or study type and biologic number**. **(a) **Bar graphs showing the percentage of patients achieving the American College of Rheumatology ACR20, ACR50 or ACR 70 criteria (see description of these criteria in Figure 1 legend), as well as remission (DAS28 <2.6) or low disease activity (DAS28 ≤3.2) according to the number of biologic disease-modifying antirheumatic drugs (DMARDs) to which the patients were exposed and whether the drug switched to was a tumor necrosis factor (TNF)-α inhibitor or a different type of biologic agent (Other). ACR20, ACR50 and ACR70 rates are based on 8 mg/kg tocilizumab, and DAS28 <2.6 and DAS28 ≤3.2 are based on abatacept. **(b) **Bar graphs showing the percentage of patients achieving ACR-based improvement criteria according to the number of biologic DMARDs to which the patients were exposed and type of study design. RCT, randomized controlled trial; Observational, observational study.

To examine whether the relationship between the number of previous TNF-α inhibitors and response rates was different for TNF-α inhibitors when compared to other types of biologic drugs (abatacept, rituximab or tocilizumab), stratified response rates were evaluated for these two main drug groups on the basis of ACR response rates and DAS28 rates of low disease activity and remission. It should be noted that the ACR response rates for the "Other" biologics group shown in Figure 2a are based on a single study of tocilizumab and that the DAS-based rates are based on a single trial of abatacept, limiting the value of the comparisons.

The results shown in Figure 2a suggest that the previously observed trend of declining response rates with increasing number of prior TNF-α inhibitors used persists for both TNF-α inhibitors and alternative biologic drugs. However, the ACR20 response rates were 54%, 42% and 14% for the second, third and fourth TNF-α inhibitors used, respectively, whereas for tociluzimab, the ACR20 rates were 49%, 50% and 54%, respectively. For TNF-α inhibitors, DAS28-based response rates for the third TNF-α inhibitor were approximately 10 percentage points lower than the rates for the second TNF-α inhibitor, but this decline was only about three percentage points in the alternative biologic drugs group (abatacept). Overall, a decline in response for the second versus the third biologic drug was generally reported for both TNF-α inhibitors and biologic DMARDs with other modes of action. However, the decline in response rates tended to be more pronounced for TNF-α inhibitors.

### Efficacy estimates stratified by study design

Several authors have reported that efficacy estimates of TNF-α inhibitors in patients naïve to biologic treatment are consistently different in RCTs and observational studies [59,60]. To determine whether the relationship between response rates and previous exposure to TNF-α inhibitors was preserved within the type of study design, estimates were also obtained after stratifying across two main types of studies: RCTs and observational studies. As shown in Figure 2b, the trend was essentially the same across the two types of study design, although response rates based on RCTs tended to be lower than those of observational studies. We examined the role of several factors which could have potentially influenced this result by comparing the characteristics of the nine observational studies [15,17,30,34,36,37,47,53] with those of the three RCTs [14,32,52] used to derive the ACR-based response rates shown in Figure 2b. These comparisons revealed that the two sets of studies were mostly similar with respect to mean age (between 45 and 54 years of age for the RCTs, and between 47 and 58 years of age for the observational studies), disease duration (between 10 and 13 years for the RCTs, and between 9 and 17 years for the observational studies) and time of efficacy assessment. (Two (67%) of three RCTs and six (67%) of nine observational studies reported efficacy at 3 and 4 months, while the remainder reported response rates at 6 and 12 months.)

Further comparisons between RCTs and observational studies indicated that whereas all nine observational studies examined the efficacy of TNF-α inhibitors (adalimumab, etanercept or infliximab), the three RCTs assessed the efficacy of golimumab, infliximab and tocilizumab. The infliximab RCT [14] used a small sample of 27 patients and hence contributed relatively little to the weighted ACR response rates. The two other RCTs had similar, much larger sample sizes, but golimumab response rates were substantially lower than those of tocilizumab. For example, the golimumab ACR20 rates for patients previously exposed to either one or two TNF-α inhibitors were both 38%, while the comparable tocilizumab ACR20 response rates were 49% and 50%. Studies which did not report response rates by the actual number of previously attempted TNF-α inhibitors were excluded from the comparison shown in Figure 2b. (This group is shown as "2+" in Figure 1.) A total of three RCTs [39,41,52] were excluded. The estimated ACR20, ACR50 and ACR70 rates based on these trials were 53%, 24% and 12%, respectively (see also Table 2 for further details), which are within the ranges formed by the response rate estimates of the second and third TNF-α inhibitors.

Comparisons of the two sets of studies with respect to the reason for discontinuation were made difficult by inconsistent reporting. In the set of RCTs, only the golimumab trial [52] reported that lack of efficacy was a reason for discontinuation of TNF-α inhibitor therapy for 58% of study participants, while 53% of participants also stated reasons unrelated to efficacy. For the infliximab [14] and tocilizumab trials [32], the reasons for discontinuation were either absent or not clearly stated. Six of the nine observational studies included patients who had discontinued therapy with a TNF-α inhibitor for reasons other than inefficacy, with study percentages ranging between 12% (5 of 41 patients) and 100% (37 of 37 patients). For observational study ACR20 response rates, for example, we estimated that approximately one-fourth of the total sample (396 of 1,512 patients) had discontinued TNF-α inhibitor therapy for reasons other than lack of efficacy. Overall, differences in reporting made it impossible to assess whether the reason for discontinuation could have explained the lower response rates observed among RCTs.

### Efficacy estimates based on reason for discontinuation

The association between response rates and reasons for discontinuation was evaluated by examining studies that reported clinical response rates to a second TNF-α inhibitor by reason for discontinuation of a first TNF-α inhibitor. These studies were selected and weighted rates were evaluated as described above and in Table 3. A total of 12 publications [16,27-29,33-38,50,51,53] provided these rates for one or more of the following groups according to reason for discontinuing a first TNF-α inhibitor: (1) lack of efficacy (patients who never achieved a response, also referred to as primary failures), (2) loss of efficacy (patients who experienced a response but lost this response over time, also referred to as secondary failures) and (3) intolerance and/or adverse events (also referred to as safety failures).

**Table 3:** Information used to evaluate the proportion of patients achieving a response to a second biologic DMARD according to common criteria used in RA studies by reason for discontinuation of a first TNF-α inhibitor^a^

Measure	Reason for discontinuation	Total number of patients	Estimated response rate, %	Biologics used to derive rate	Treatment duration range, months	Mean RA duration range, yr	Reference sources
ACR20	Intolerance	337	62.5	ADA and TNF_2_	3	12	[17,36,51,53]
	Lack of efficacy	251	48.4	ADA and ETN	3 to 4	9 to 12	[34,36,37,53]
	Loss of efficacy	609	58.0	ADA and ETN	3 to 6	9 to 12	[30,34,36,37,53]
ACR50	Intolerance	337	35.7	ADA and ETN	3	12	[17,36,51,53]
	Lack of efficacy	251	23.6	ADA and ETN	3 to 4	9 to 12	[34,36,37,53]
	Loss of efficacy	537	29.6	ADA and ETN	3 to 4	9 to 12	[34,36,37,53]
ACR70	Intolerance	337	13.4	ADA and TNF_2_	3	12	[17,36,51,53]
	Lack of efficacy	251	9.0	ADA and ETN	3 to 4	9 to 12	[34,36,37,53]
	Loss of efficacy	537	12.0	ADA and ETN	3 to 4	9 to 12	[34,36,37,53]
DAS28 <2.6	Intolerance	443	15.2	ABA and TNF_2_	3 to 6	NR	[17,29]
DAS28 <3.2	Intolerance	211	30.4	TNF_2_	3 to 6	6	[17,35]
	Lack of efficacy	98	13.0	TNF_2_	3 to 6	6	[35]
	Loss of efficacy	150	12.0	TNF_2_	3 to 6	6	[35]
EULAR moderate	Intolerance	250	38.8	TNF_2_	3 to 6	9	[38,45,50]
	Lack of efficacy	98	37.0	TNF_2_	3 to 6	6	[35]
	Loss of efficacy	150	18.0	TNF_2_	3 to 6	6	[35]
EULAR good	Intolerance	718	21.3	ADA and TNF_2_	3 to 12	6 to 12	[17,35,36,38,45,50,53]
	Lack of efficacy	320	15.2	ADA, ETN and TNF_2_	3 to 6	6 to 12	[35-37]
	Loss of efficacy	515	16.9	ADA, ETN and TNF_2_	3 to 6	6 to 12	[35-37]
EULAR moderate/good	Intolerance	467	69.5	ADA and TNF_2_	3 to 6	6 to 12	[17,35,36,45,53]
	Lack of efficacy	349	63.2	ADA, ETN and TNF_2_	3 to 6	6 to 12	[34-37,53]
	Loss of efficacy	687	60.7	ADA, ETN and TNF_2_	3 to 6	6 to 12	[34-37,53]

^a^DMARD, disease-modifying antirheumatic drug; RA, rheumatoid arthritis; ACR, American College of Rheumatology; DAS28, Disease Activity Score 28 joint count; EULAR, European League Against Rheumatism; ABA, abatacept, ADA, adalimumab, ETN, etanercept, TNF_*i*_, *i*th TNF-α inhibitor; ABA, abatacept; ADA, adamlimumab; TNF_*i*_, *i*^th ^TNF-α inhibitor. ACR20 means a 20% improvement in tender or swollen joint counts as well as 20% improvement in at least three of the following five criteria: patient assessment, physician assessment, erythrocyte sedimentation rate, pain scale and functional questionnaire. The ACR50 and ACR70 categories adhere to the same criteria, but for 50% and 70% improvement, respectively.

Five publications which were initially identified for potential inclusion in these analyses had to be excluded because of problems in the way this information was reported. Two studies [49,61] were excluded because only change in continuous outcomes (HAQ and DAS28) was reported. One study [58] which reported the effects of adalimumab treatment among TNF-α inhibitor inadequate responders was excluded because the length of the period leading up to assessment of response varied between 1 and 19 months, making it difficult to interpret the published rate. One study [52] was excluded because it reported ACR response rates according to whether the reason for discontinuation was related to efficacy, which did not fit with the classification defined above. A fifth study [47] was excluded because its eligibility criteria limited the patient sample to those who responded to treatment with infliximab and switched to etanercept as a result of adverse events. We find that this particular group of patients is likely not comparable to those included in the remaining studies, since for other studies patients were classified as safety failures because this was the primary reason for discontinuation, regardless of whether they had experienced an effective response to the first TNF-α inhibitor.

**Figure 3:**
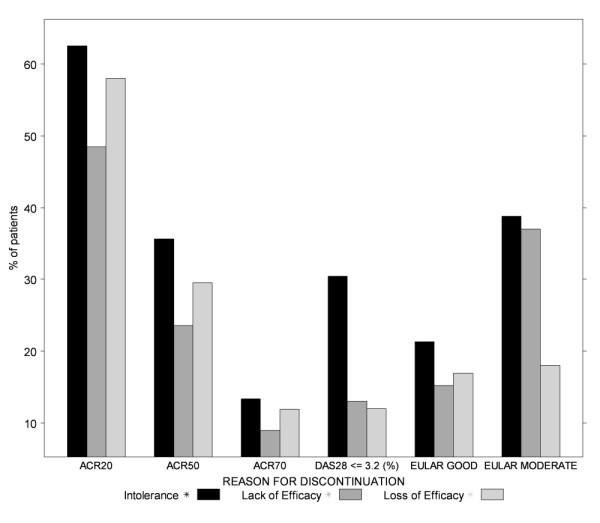
**Percentage of patients responding to a second TNF-α inhibitor by reason for discontinuation of first**. Bar graph showing the percentage of patients achieving a response to a second tumor necrosis factor (TNF)-α inhibitor according to criteria commonly used in rheumatoid arthritis by reason of discontinuation of the initial TNF-α inhibitor. ACR, American College of Rheumatology (see Figure 1 legend for description of ACR20, ACR50 and ACR 70 criteria); DAS28, Disease Activity Score 28 joint count; EULAR, European League Against Rheumatism.

Figure 3 presents the weighted ACR, DAS28 and EULAR rates of response for a second TNF-α inhibitor by reason for discontinuation of an initial TNF-α inhibitor. It should be noted that DAS28-based remission (DAS28 <2.6) rates were reported only for safety failures, making it impossible to compare the three groups based on this response criterion. Response rates for primary versus secondary failures were not consistently ordered across the six response criteria shown in Figure 3. On the basis of ACR response rates, secondary failures appear to have a greater likelihood of responding to a second TNF-α inhibitor. Contrary to this finding, rates of EULAR moderate response suggested that a greater proportion of primary failures would respond to a second TNF-α inhibitor compared to secondary failures. The two remaining response criteria (EULAR good and DAS28 ≤3.2) are generally too close to suggest a clear difference between these two groups.

Two key differences may help explain the discrepancies in these results. First, the study follow-up (or time to assessment of response) tended to be somewhat longer in the studies in which rates of DAS-based remission and rates of EULAR moderate response were derived than in studies used to derive ACR and good EULAR response rates. Second, these estimates were often based on a few studies with total sample sizes that ranged between 98 and 250 patients. In contrast, ACR response rates and EULAR good response rates were each estimated on the basis of four or more studies with total sample sizes that ranged between 251 and 609 patients, making the ACR and EULAR good response estimates somewhat more robust. Nearly 50% and 60% of primary and secondary failures, respectively, were estimated to achieve an ACR20 response. Overall, ACR response rates were approximately 20% to 30% higher for secondary failures when compared to those of primary failures.

In contrast to the results obtained for primary and secondary failures, response rates to a second TNF-α inhibitor were consistently higher among patients who switched for safety-related reasons. More than 60% of safety failures were estimated to reach an ACR20 response, and about one-half (51%) were estimated to experience a EULAR moderate response; approximately one-third of these patients achieved an ACR50 response and low disease activity (DAS28 ≤3.2).

### Efficacy estimates stratified by length of follow-up

Since the ideal period for determining whether a response to treatment has occurred remains controversial [62] and varied considerably across the selected studies, we examined how efficacy varied by length of follow-up or time of efficacy assessment. Differences in ACR20 response rates for the second versus third biologic drug used were similar for treatment durations of 3 to 4 months and durations of 6 months or more (about 10% in both cases).

## Discussion

In the current study, the association of response to subsequent biologic treatment with number of previous TNF-α inhibitor treatments was evaluated on the basis of data reported in peer-reviewed publications. After combining these data, the results indicated that an association does in fact appear to exist and that response is likely to decline with increasing number of previous TNF-α treatments. Our results also suggest that the pattern of decreasing response for increasing number of failed TNF-α inhibitors was maintained even when the analyses were restricted to more homogeneous groups of studies. Importantly, we found that the relationship was also maintained across a number of important RA response measures, which further contributes to the validity of the findings. Furthermore, the ACR response rates derived in the current study for patients with no prior exposure to biologic drugs are in line with those reported in previous studies that examined 6-month outcomes from RCTs using patients previously not exposed to biologic DMARDs [10].

One limitation of the current study is that the degree of heterogeneity across studies was not summarized quantitatively. Nevertheless, it must be clear from our exposition regarding the difficulties in combining results across studies that a large degree of variation in study design and patient characteristics was present. In addition, the results that were central to our research question were frequently incidental to the primary objectives of the studies that were reviewed, which meant that information specific to groups of patients differing in the number of prior TNF-α inhibitors used was often lacking. Consequently, no formal statistical inference was undertaken, which is another limitation of the study.

Despite having found several results supporting a trend of lower efficacy rates with increased number of previous TNF-α inhibitors used, we believe that more research into this topic is needed before a conclusion can be reached. In particular, the safety of biologic treatment specifically in patients with an inadequate response to TNF-α inhibitors must also be addressed before the strategy of switching can be fully evaluated vis-à-vis alternative therapies.

One potential key factor in predicting response after a switch to an alternative biologic drug is the reason for discontinuation of the prior drug. The current study examined this aspect of RA treatment within the scope of the available data on this topic, which did not enable a comparison of patients who switched to a second biologic DMARD that was not within the TNF-α inhibitor class. Although several studies did report reasons for discontinuation of prior TNF-α inhibitor treatment, only a few reported response rates for groups of patients who differed in the reason for discontinuation. Our results indicate that patients who discontinue treatment as a result of adverse events are more likely to achieve a clinical response to a second TNF-α inhibitor than are patients who discontinue the first TNF-α inhibitor for efficacy-related reasons. Higher response rates [30] and greater declines in DAS28 values [49] among safety failures have been reported in other studies. Nevertheless, some caution should be taken when interpreting these findings, since the assessment of these rates could be sensitive to the length of follow-up in a particular study. Two large studies based on registry cohorts [16,45] reported that the reason for discontinuation of a first TNF-α inhibitor was likely to explain the reason for discontinuation of a second TNF-α inhibitor, while the findings from another large registry cohort could not confirm this relationship [50]. Further, response rates in a RCT of the newer biologic agent tocilizumab [52] were nearly identical for patients who discontinued treatment because of inefficacy or for unrelated reasons. Overall, we observed consistently higher response rates among safety failures than among primary and secondary efficacy failures. Data obtained over longer follow-up periods, however, are needed to confirm or disprove the findings of the current study.

The exact form of the relationship between response to treatment and number of failed TNF-α inhibitors is likely to play an increasingly important role in defining treatment strategies for RA patients who have had an inadequate response to treatment with TNF-α inhibitors. Given the high cost of biologic agents and evolving knowledge of their safety profiles, there is a growing need to compare and evaluate the relative benefit of strategies involving alternative sequences of therapies. The recent availability of a number of newer biologic drugs with different mechanisms of action makes this need even more salient. Establishing patterns of response along the treatment pathway is a key element of these evaluations, as is the identification of subgroups of patients who may differ in these response patterns. In addition to providing insight into the existence of an association between treatment response and increasing number of failed TNF-α inhibitors, the current study has also offered an overview of the multiple difficulties that are faced when synthesizing evidence pertaining to this question. Differences in reporting, study design and the overall availability of data have made this task a difficult one, which is apparent from the results presented herein.

Given that a substantial proportion of patients will fail an initial biologic treatment, establishing when and how to initiate treatment with these agents is just as important as establishing the relative value of long-term treatment strategies. In many instances, these long-term strategies involve a sequence of treatments, so the question arises at each step which therapy to use as a replacement when a particular therapy must be stopped because of inefficacy or intolerance. Multiple studies have addressed this question in a number of different ways. To date, the large majority consist of observational studies. Some of the results of earlier observational studies were based on small samples and relatively short follow-up periods [29,44,46,51], but more recently the accumulation of data from several registries of RA patients [61] treated with biologic drugs has provided larger sample sizes and longer duration of follow-up. In addition, while the older biologic DMARDs were compared primarily with nonbiologic DMARDs, RCTs of newer biologic drugs have used samples of patients with an inadequate response to one or more biologic DMARDs, primarily TNF-α inhibitors [63]. Several reviews [64-68] have attempted to summarize data from these studies, but no clear guidance has emerged from these publications. A recent meta-analysis [69] presented a quantitative evaluation of the effectiveness of switching treatments, specifically between TNF-α inhibitors. These results suggested that the probability of achieving a clinical response declines after the first TNF-α inhibitor, a trend that was observed in our study as well, even when other biologic DMARDs were considered. Although data were limited, we found that the magnitude of this decline may depend on the type of biologic drug administered. For example, DAS28-based results suggested that when the third biologic is a TNF-α inhibitor, response rates may be lower than those of an alternative biologic. Although readers are cautioned to consider the limitations of the available data and the preliminary nature of the findings, our study strengthens and extends the findings presented in the meta-analysis of Lloyd *et al. *[69].

Establishing the existence and magnitude of a relationship between responses to biologic drug treatment and the number of previously failed TNF-α inhibitors has a number of important implications. From a clinical practice perspective, understanding such a relationship can help clinicians to decide on a treatment to use from among an increasing number of alternative strategies. From a research perspective, the current investigation may help inform future studies that involve the treatment of RA patients over the course of their lifetimes, such as economic assessments of RA therapies based on treatment sequence models. One of the key objectives of this work was to provide a more quantitative analysis of the data reported in studies of patients refractory to TNF-α inhibitors, something that was lacking in the current literature. In addition, the current study has exposed some of the difficulties associated with combining results across studies with different designs, patient populations and reported outcome measures. Exposing these limitations may improve the design of future research and foster greater harmonization of RA clinical studies that aim to investigate the effects of sequential biologic drug therapies in RA.

## Conclusions

For patients with prior exposure to TNF-α inhibitors, the likelihood of a response to subsequent treatment with biologic agents declines with an increasing number of previous TNF-α inhibitor treatments.

## Abbreviations

ABA: abatacept; ACR: American College of Rheumatology; ADA: adalimumab; ANA: anakinra; CRP: C-reactive protein; DAS28: Disease Activity Score 28 joint count; DMARD: disease-modifying antirheumatic drug; ESR: erythrocyte sedimentation rate; ETN: etanercept; EULAR: European League Against Rheumatism; GLM: golimumab; HAQ: Health Assessment Questionnaire; IFX: infliximab; LEF: leflunomide; MTX: methotrexate; NICE: National Institute for Clinical Excellence; RCT: randomized controlled trial; RTX: rituximab; TCZ: tociluzimab; TNF: tumor necrosis factor; VAS: Visual Analog Scale.

## Competing interests

GVW, TK, JB and SHZ are employees of Pfizer Inc., the sponsor of this study. RRB, MY and MK have served as consultants for Pfizer.

## Authors' contributions

RRB and GVW performed the literature search and data analysis and wrote and edited the manuscript. TK, JB and SHZ assisted with the editing of the manuscript, the subject matter content and the conclusions. MY and MK assisted with the development of the manuscript outline, the conceptualization of the study and data interpretation. All authors were involved in discussion of the findings as well as in the drafting and final approval of the manuscript.

## Supplementary Material

Additional file 1 **Table S1. Characteristics and main findings of studies included in the analyses**. Description: Microsoft Word table containing the following information for each of the 28 studies included in the analyses: study publication(s); study design; anti-tumor necrosis factor (anti-TNF) groups (anti-TNF naïve, first-, second- or third-time switchers and the biologics involved); disease duration; mean age, baseline DAS28; duration of follow-up (months); and key study findings. Click here for file
