# Supplementary material for: Evaluating the efficacy of sequential biologic therapies for rheumatoid arthritis patients with an inadequate response to tumor necrosis factor-α inhibitors
Source: Arthritis Res Ther. 2011 Feb 16;13(1):R25. doi: 10.1186/ar3249 (PMC3241369; doi:10.1186/ar3249)
Supplement: Additional file 1 — Table S1. Characteristics and main findings of studies included in the analyses. Description: Microsoft Word table containing the following information for each of the 28 studies included in the analyses: study publication(s); study design; anti-tumor necrosis factor (anti-TNF) groups (anti-TNF naïve, first-, second- or third-time switchers and the biologics involved); disease duration; mean age, baseline DAS28; duration of follow-up (months); and key study findings. [file ar3249-S1.DOC]

**Table A** Characteristics and main findings of studies included in the analyses

| *Source* | *Design* | *Anti-TNF groups* | *Disease Duration*  *(years)* | *Mean Age* | *Baseline*  *DAS28* | *Duration of*  *follow-up (months)^(3)^* | *Results* |
| --- | --- | --- | --- | --- | --- | --- | --- |
| Furst 2007*‡[14] | Randomized, open-label study of patients with inadequate response to ETN; | Switchers (ETN-IFX; N=13) | 10 | 45 | 6.2 | 4 | Results based on ACR/DAS28 criteria suggested greater improvement among patients who switched to IFX when compared to those who continued ETN; no formal statistical inference due to small sample size. |
| Haraoui 2004*[15] | Prospective open-label study | Switchers (IFX-ETN; N=22) | 11 | 50 | - | 3 | ACR20 and HAQ improved in more than 60% of patients. Only 22 (out of 25) completed 12 week assessment; 19 discontinued IFX because of inefficacy (ACR20). Mean duration of therapy with IFX was 15.7 months. |
| Karlsson 2008*‡ [17]  SSTAG | Prospective observational study (registry). | Second-time switchers (N=36) | 15 | 58 | 5.8 | 3 | **ACR and EULAR response criteria showed lower levels of improvement for second time switchers when compared to those patients switching for the first time.** |
|  |  | First-time switchers (N=337). | 14 | 56 | 5.5 |  |  |
| Schiff 2009[29]  ARRIVE | Open-label trial of patients previously treated with TNF-α inhibitors | Third-time switchers (TNF_3_-ABA; N=200)  Second-time switchers (TNF_2_- ABA; N=340)  First-time switchers (TNF_1_- ABA; N=488) | 12^(6)^ | 54^(6)^ | 6.2^(6)^ | 6 | **There was trend of lower DAS28-based response criteria for higher number of previous TNF-α inhibitors, particularly for 3 or more.** Percentage achieving low disease activity was 25%/23% 15% for 1/2/3 prior TNF-α inhibitors. Percentage achieving remission was 16%/13% 7% for 1/2/3 prior TNF-α inhibitors. Whether patients had undergone washout or were switched directly to ABA after stopping TNF-α inhibitor treatment did not influence improvement. |
| Wick 2005*‡[30] | Retrospective study of 62 patients treated with ADA with secondary loss of efficacy; | Switchers (IFX-ADA; N=27). | - | 50 | 5.5 | 6 | ACR20 was 70%/75% for patients who switched from IFX/ETN and 70% for anti-TNF-naïve patients. Results suggested similar effect of ADA for patients experiencing secondary loss of efficacy and those with no prior anti-TNF exposure.  Fifty-nine (95%) of the 62 patients had RA. |
|  |  | Switchers (ETN-ADA; N=9). | - | 47 | 6.6 |  |  |
|  |  | TNF-naïve (ADA; N=26). | - | 53 | 5.6 |  |  |
| van Vollenhoven 2003[31]  STURE/ARTIS | Prospective observational study. | Switchers (ETN-IFX; N=18)  Switchers (IFX-ETN; N=13) | 15  15 | 53  49 | 5.9  6.4 | 3 | Improvement in joint counts appears greater in IFX-ETN group. ETN/IFX was taken for an average of 6.8/5.5 months before discontinuation. The reason for switching was primarily inefficacy (14/18) in ETN-IFX group, and AEs (11/13) in IFX-ETN group. The proportion of patients taking MTX was increased during the second treatment in the ETN-IFX group, but it was decreased in the IFX-ETN group. |
| Emery 2008*[32]  RADIATE | RCT of patients with an inadequate response to one or more TNF inhibitors. | Third-time switchers (TNF_3_-TCZ; 8 mg: N=26; 4 mg: N=18; PBO: N=18)  Second-time switchers (TNF_2_-TCZ; 8 mg: N=52; 4 mg: N=60; PBO: N=64)  First-time switchers (TNF_1_-TCZ; 8 mg: N=92; 4 mg: N=81; PBO: N=76) | 11-13 ^(1)^ | 51-54^(1)^ | 6.8^(1)^ | 6 | **Results suggest no clear dependence of ACR20/50/70 by previous number of anti-TNFs treatments. For TCZ arm (8mg), ACR20/50/70 was 49%/50%/ 54% for first/second/ third time switchers.**  Patients discontinued ETN (≥ 2 weeks), IFX/ADA (≥8 weeks), LEF (≥12 weeks); stable MTX (≥8 weeks). |
| Bennett 2005[33] | Prospective observational study | Switchers (IFX/ETN/ANA-ADA; N=26)  TNF-naïve (ADA;  N=44). | -  - | -  - | 6.2  6.2 | 8.5  8 | The improvements from baseline in DAS28 and HAQ were statistically significant for both anti-TNF-naïve and exposed group. When comparing between groups the change in DAS28 was only marginally significant (P=0.069) and change in HAQ was not significant (P=0.88).  The mean duration of previous biologic treatment was 9 months (range 0.5–24). The reasons for changing were: secondary failure (to IFX only) in 45% (13) of cases, primary failure in 27% (8) and adverse events in 21% (6). |
| Bingham III 2009*‡[34] | Open-label trial of subjects with lack or loss of response to IFX | Switchers (IFX–ETN; N=201)  . | 9 | 57 | 6.6 | 4 | ACR20/50/70 was 42%/18%/8% after 4 months of ETN treatment. No washout period between IFX and ETN.  Response to ETN was independent of whether improvement had ever been achieved with IFX or was achieved and subsequently lost. |
| Blom 2009‡[35] | Prospective observational study | Switchers (TNF_1-_ TNF_2_;N=197) | 8 | 55 | 5.1 | 6 | One third of patients achieved moderate or good EULAR response at 6 months. No significant differences were found in outcomes by reason for discontinuation (lack of response, loss of response or intolerance) |
| Bombardieri 2007*‡[36] ReACT | Open-label trial of patients treated with ADA. | Second-time switchers (ETN and IFX – ADA; N=120) | 12 | 52 | 6.6 | 3 | **ACR and EULAR response criteria showed lower levels of improvement for patients with exposure to both ETN and IFX (N=120) when compared to patients who had been treated with only one of these agents (N=779).**  Discontinuation of biologics ≥ 2months prior to study entry.  No significant increase in AEs among patients who had received previous anti-TNF treatment when compared to anti-TNF naïve patients. |
|  |  | First-time switchers (IFX-ADA; N=591) | 12 | 53 | 6.2 |  |  |
|  |  | First-time switchers (ETN-ADA; N=188) | 13 | 54 | 6.5 |  |  |
|  |  | TNF-naïve (ADA; N=5,711). | 11 | 54 | 6.0 |  |  |
| Buch 2007*‡[37] | Retrospective study of patients who had failed IFX and subsequently switched to ETN. | Switchers (IFX-ETN; N=72). | - | 57 | 6.4 | 3 | ACR20/50/70 after 6 months of ETN treatment was 38%/24%/15%. Results suggested that outcomes (ACR/DAS28) of ETN treatment are better among patients who never achieved a response (ACR20) than those who achieved a response but subsequently lost it.  Primary non-response patients took IFX for 3 months; loss of efficacy group took IFX for a median of 60 weeks (range 24–216 weeks). |
| Caporali 2009 ‡[38]  LORHEN | Prospective observational study (registry). | Switchers (TNF_1-_ TNF_2_;N=237) | 8 | 61 | 5.7 | 12 | More than two-thirds of patients achieved moderate or good EULAR response at 12 months. Patients who started treatment with a second TNF-α inhibitor due to lack of response were more frequently responders than those who started the second treatment because of adverse events. |
| Cohen 2006[39];  Keystone 2009[48]  REFLEX | RCT of patients with an inadequate response to one or more TNF inhibitors. | Switchers (TNF_1+_-RTX; N=298) | 12 | 52 | 6.9 | 6 | Results strongly suggest improvement based on ACR/EULAR criteria and several patient-reported outcome (PRO) measures (ACR20/50/70=51%/27%/12% and EULAR low disease/remission = 15%/9%, in RTX group). Results not reported by number of previous anti-TNFs.  Percentage of patients who had failed 1, 2 and 3 anti-TNFs was 60%, 31% and 9%, respectively. MTX (10-25 mg/week) ≥12 wks before screening; inadequate response to anti-TNF for ≥ 3 months; patients discontinued ETN (≥ 4 weeks), IFX/ADA (≥ 8 weeks). |
|  |  | Stoppers (TNF_1+_-PBO; N=201) | 12 | 53 | 6.8 |  |  |
| Finckh 2007[40]  swiss clinical quality management RA | Prospective observational study | Switchers (TNF_1/2_-RTX; N=50).  Switchers (TNF_1/2_- TNF_2/3_; N=66). | 10  9 | 50  66 | 5.4  5.0 | 18 | **Evolution of the DAS28 was more favorable in the group that received RTX than in the group that switched to an alternative anti-TNF agent.** **The predicted mean change in DAS28 was +0.44 for patients with > 1 previous anti-TNF trials (P=0.05 versus previous failure with 1 anti-TNF).** |
| Genovese 2005[41]; Genovese 2008[42]  ATTAIN | RCT of patients previously treated with ETN/IFX, with an 18 month open-label phase. | Switchers (IFX/ETN –ADA; N=256) | 12 | 53 | 6.5 | 24 ^(5)^ | At 6 months 50%/20% of ADA/PBO patients achieved ACR20. At 12/ 24 months, 60% /56% (N=218) of patients initially randomized to ADA achieved ACR20. After 6/ 18 months of ADA, 65% /52% (N=99) of patients initially randomized to PBO achieved ACR20.  Patients discontinued ETN (≥ 4 weeks) and IFX (≥8 weeks); stable oral DMARD or ANA for ≥ 4 weeks. 62% of patients were no longer receiving ETN/IFX at time of screening. 75%/82% of ADA/PBO arm was taking MTX. |
|  |  | Stoppers (IFX/ETN-PBO; N=133) | 11 | 53 | 6.5 |  |  |
| Gomez-Puerta 2004[43] | Prospective observational study. | Switchers (IFX-ETN; N=12). | - | - | 5.6 | 6 | Results suggest good EULAR response rates after ETN; IFX had been administered for a mean of 15.6 months (range 2-29). |
| Hansen 2004[44] | Retrospective study/chart review; | Switchers (ETN – IFX; N=20) | 9 | 48 | - | 3 | Clinical improvement was similar across groups, but switchers received higher dose of IFX. All patients were taking LEF at study entry.  The reason for discontinuation for the second anti-TNF was mildly related to the reason for discontinuation of the first: 70% of those who discontinued (D/C) the second anti-TNF because of lack of efficacy also D/C the first because of lack of efficacy; for D/C AEs this number was ~ 60%. |
|  |  | TNF-naïve (IFX; N=73) | 11 | 54 |  |  |  |
|  |  | Stayers (ETN; N=14). | 12 | 52 |  |  |  |
| Hjardem 2007‡[45]  DANBIO | Observational study of patients treated with ≥ 2 anti-TNFs. | Switchers (N=156). | 8 | 55 | 5.9 ^(2)^ | 3 | **For switchers response after 3 months of treatment was similar for first (mean DAS28 Δ = -1.2) and second (mean DAS28 Δ = -1.0) TNF-α inhibitor**. Effect of second TNF-α inhibitor was greater when reason for switching was lack of efficacy. The sample sizes differ substantially across time points and for different measures making the results difficult to interpret. First drug was: 76% IFX, 17% ADA, 8% ETN. |
|  |  | Stayers (N=583) | 9 | 57 | 5.5 ^(2)^ |  |  |
| Hyrich 2008[46]  BSRBR | Retrospective study; patients who had 12 months of follow up after not responding to the first anti-TNF. | Switchers (N=331) | 11 | 58 | 6.7^(2)^ | 12 | HAQ scores changes remained essentially unchanged for patients who discontinued treatment over subsequent 12 months. Patients who continued with the same anti-TNF despite inadequate response, continued to improve, albeit at a lower rate than patients who switched to an alternative anti-TNF.  Patients were selected based on reason for stopping being inefficacy or according to EULAR criteria (assessed at 6 and 12 months after starting first anti-TNF; 88% were classified as non-responders at 6 months). Patients who discontinued because of AEs were excluded. |
|  |  | Stayers (N=389) | 12 | 58 | 6.3^(2)^ |  |  |
|  |  | Stoppers (N=148). | 12 | 61 | 6.6^(2)^ |  |  |
| Iannone 2007*[47] | Retrospective study of patients who were successfully treated with IFX but switched to ETN because of Aes. | Switchers (IFX-ETN; N=37). | 8 | 49 | - | 12 | Greater percentage of patients achieving ACR50/70 (73%/50%) after 6 months of ETN, when compared to ACR50/70 during prior 6 months of IFX treatment; decrease in mean pain scores (VAS); no significant changes in DAS44, HAQ, RAI, ESR and CRP, from scores 8 weeks prior to starting ETN.  Time between IFX and ETN was 2-4 weeks. IFX outcomes were not measured after 6 months of treatment, rather they were measured 6 months prior to the switch, making it difficult to compare. |
| Laas 2008[49] | Prospective observational study. | Switchers – inefficacy (IFX-ETN; N=20).  Switchers – AEs (IFX-ETN; N=6).  Switchers – Non-medical (IFX-ETN; N=23). | 8  12  16 | 51  56  50 | 5.5  5.1  2.6 | 9 | DAS28 change scores suggest improvement for inefficacy and AEs group and stable response for non-medical. Follow-up for each group is not reported (overall mean follow-up was 16 months). Results noted refer to values taken from Figure 2 in the publication, which are nevertheless similar to those reported for the last follow-up visit. Duration of IFX treatment varied substantially across groups: 16 months for inefficacy, 6 months for Aes and 27 months for non-medical. |
| Navarro-Sarabia 2009‡[50] | Prospective cohort study; | Second-time switchers (TNF_3_; N=18) | 10 | 44 | 6.1 | 8.5 | Results suggest no improvement over start of therapy for patients who switch to a 3^rd^ anti-TNF. EULAR non-response rates were 25%, 53% and 72% for first, second and third anti-TNF, respectively. **Statistically significant improvement in HAQ/DAS28 scores for first and second but not for third anti-TNF.**  EULAR response was similar for patients switching to a second anti-TNF due to inefficacy (55%) or AEs (45%). Comparison groups are not mutually exclusive. |
|  |  | First-time switchers (TNF_2_; N=81) | 10 | 52 | 5.1 | 9.7 |  |
|  |  | TNF-naïve (TNF_1_; N=417). | 11 | 53 | 6.1 | 17.4 |  |
| Nikas 2006‡[51] | Retrospective case-control study; | Switchers (IFX-ADA; N=24). | 17 | 57 | 5.6 | 12 | EULAR and ACR improvement criteria essentially equal for both switchers and those with no prior IFX exposure. Improvement was greater among patients who switched for efficacy reasons than for AEs, but sample sizes for these comparisons were small.  Switchers were matched for age, sex, disease duration and DAS28, and compared to patients with no previous IFX exposure; time between IFX and ADA varied between 4 and 10 weeks. Switchers had received IFX for an average of 18.5 months. |
|  |  | TNF-naïve (ADA; N=25). | 16 | 56 | 5.9 |  |  |
| Smolen 2009*[52]  GO-AFTER | RCT of patients previously treated with anti-TNF. | Third-time switchers (TNF_3_-GLM; 100/50 mg: N=22; PBO: N=21)  Second-time switchers (TNF_2_- GLM; 100/50 mg: N=71; PBO: N=44)  First-time switchers (TNF_1_- GLM; 100/50 mg: N=213; PBO: N=90) | 9-10^(1)^ | 54-55^(1)^ | 6.1-6.3^(1)^ | 3.5^(4)^ | **Results suggest no difference in ACR20 after TCZ treatment for patients who were previously treated with 1 (38%) or 2 (38%) anti-TNFs. Patients treated with 3 anti-TNFs had lower ACR20 rates (14%).**  For patients who had received 1 TNF-α inhibitor, achievement of ACR20 was independent of previous drug. Reason for discontinuation did not appear to influence ACR20 rates. Patients discontinued ETN (≥ 2 weeks), IFX/ADA (≥8 weeks), LEF (≥12 weeks); stable MTX (≥8 weeks). |
| van der Bijl 2008*‡[53] | Prospective open-label study. | Switchers (IFX-ADA; N=41). | 12 | 55 | 6.1 | 4 | Meaningful improvements in all measures, with greater response rates observed for patients who had experienced loss of initial response or intolerance of IFX. |

**Study included in subset used to derive estimates shown in Figure 2.a (response rates by type of study design)*

*‡Study included in subset used to derive estimates shown in Figure 3 (response rates by reason for discontinuation)*

*^(1)^ Values not reported by previous TNF exposure group; results shown are averages across treatment arms.*

*^(2)^ Median.*

*^(3)^ Duration of follow up reflects the time for assessment of most recent treatment, not the total duration of the study.*

*^(4)^ At week 16, patients who had less than 20% improvement in both tender and swollen joint counts were eligible to receive 50/100 mg if in they were randomized to the placebo/50 mg group. Total trial duration was 24 weeks.*

*^(5 )^ Genovese 2005 reported 6-month outcomes (sample sizes as shown above), while Genovese 2008 reported 12 and 24 months outcomes.*

*^(6)^ Value not reported by previous number of TNF-α inhibitor treatments; values are for overall study sample.*

*Abbreviations: ABA = abatacept, ADA = adalimumab, AE = adverse event, ANA = anakinra, CRP = C-reactive protein, D/C = discontinued, DAS = Disease Activity Score, ESR = Erythrocyte sedimentation rate, ETN = etanercept, GLM = golimumab, HAQ = Health Assessment Questionnaire, IFX = infliximab, LEF = leflunomide, MTX = methotrexate, RAI = Ritchie Articular Index, RTX = rituximab, TNF_i_ = i^th^ TNF-α inhibitor, TCZ = tocilizumab, TNF = Tumor necrosis factor, VAS = visual analog scale.*
